# The impact of economic downturns and budget cuts on homelessness claim rates across 323 local authorities in England, 2004–12

**DOI:** 10.1093/pubmed/fdv126

**Published:** 2016-10-17

**Authors:** Rachel Loopstra, Aaron Reeves, Ben Barr, David Taylor-Robinson, Martin McKee, David Stuckler

**Affiliations:** 1 Department ofSociology, Oxford University, Oxford OX1 3UQ, UK; 2 Department of Public Health & Policy, University of Liverpool, LiverpoolL69 3GB, UK; 3 Department of Public Health & Policy, London School of Hygiene & Tropical Medicine, LondonWC1H 9SH, UK

**Keywords:** austerity, homelessness, recession

## Abstract

**Background:**

It is unclear why rates of homelessness claims in England have risen since 2010. We used variations in rates across local authorities to test the impact of economic downturns and budget cuts.

**Methods:**

Using cross-area fixed effects models of data from 323 UK local authorities between 2004 and 2012, we evaluated associations of changes in statutory homelessness rates with economic activity (Gross Value Added per capita), unemployment, and local and central government expenditure.

**Results:**

Each 10% fall in economic activity was associated with an increase of 0.45 homelessness claims per 1000 households (95% CI: 0.10–0.80). Increasing rates of homelessness were also strongly linked with government reductions in welfare spending. Disaggregating types of welfare expenditure, we found that strongest associations with reduced homelessness claims were spending on social care, housing services, discretionary housing payments and income support for older persons.

**Conclusions:**

Recession and austerity measures are associated with significant increases in rates of homelessness assistance. These findings likely understate the full burden of homelessness as they only capture those who seek aid. Future research is needed to investigate what is happening to vulnerable groups who may not obtain assistance, including those with mental health problems and rough sleepers.

## Introduction

**Fig. 1:**
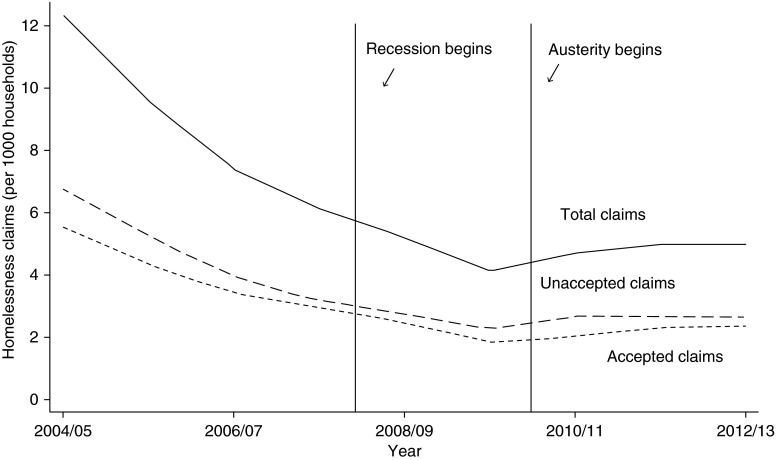
Trends in mean homelessness claim rates across 323 local authorities in England, 2004–12. *Notes:* Authors' calculations.^34^ Austerity denotes beginning of spending cuts in UK, as outlined in initial 2010 Spending Review.^22^ Accepted claims: households meeting criteria for statutory homelessness assistance from local authority. Unaccepted claims: households who applied, but did not meet criteria, for statutory homelessness assistance from local authority.

The statutory homelessness system in England, first legislated by the Housing (Homeless Persons) Act in 1977, places a duty on local authorities to secure accommodation for those making claims for homelessness assistance who meet statutory homelessness criteria. They are also required to offer other assistance to those who do not meet priority need criteria but are experiencing homelessness.^1^ After nearly a decade of decline in the number of such people, there was a reversal in 2010 when rates began to rise (Fig. 1). This is a concern for public health as a significant body of epidemiologic research demonstrates that homelessness increases risks of infectious disease, physical harm, food insecurity, multiple morbidities and premature mortality.^2–6^

**Fig. 2:**
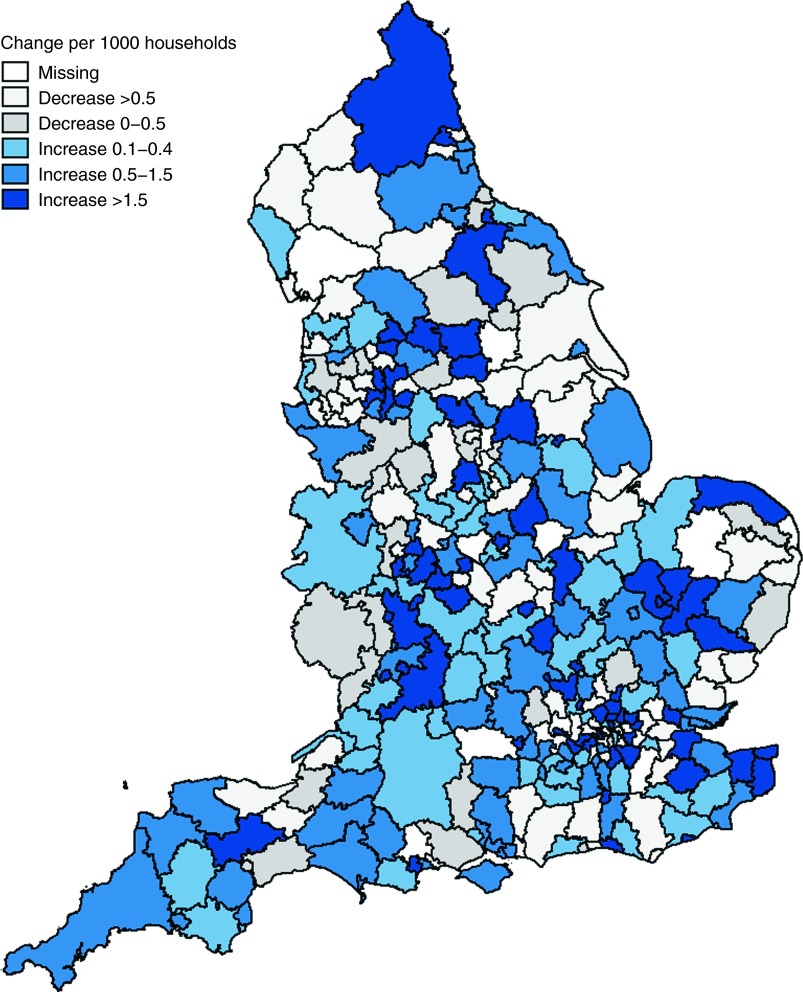
Change in homelessness claim rates between 2009 and 2012 across 323 local authorities in England.

Yet the picture varies markedly across England. Between 2009 and 2012 about one in three local authorities experienced increases of over 50% in homelessness claims, whilst another third experienced overall declines (Fig. 2).

Often viewed as a marker of wider societal distress, homelessness is frequently a consequence of extreme poverty among groups who are disabled, have histories of mental health problems and/or substance use, and whose housing arrangements are unstable.^7^ Homelessness can be ‘transitional’, intermittent, or ‘chronic’, and persons may be sheltered in temporary accommodation, facing eviction, or be unsheltered ‘rough sleepers’.^2^

Homelessness is associated with structural factors such as the affordability of housing, the coverage and size of income transfers, and the local labour market conditions.^7–11^ Much of the research on these factors has been conducted using data from US metropolitan areas. For example, studies have identified the role that unemployment rates and levels of income support play in geographical variations in homelessness prevalence.^12,13^ In contrast, there have been relatively few studies of the structural causes of homelessness in Europe using quantitative data.^11^ One study from England found that between 1992 and 2008, higher housing prices, lower household incomes and fewer homelessness prevention programmes were significantly linked to greater statutory homelessness rates.^14^

Reduced income and cuts to social safety nets such as housing support budgets are two factors that could explain why homelessness has risen so sharply in England.^15,16^ Incomes have fallen through job losses or cuts in remuneration, both in hours worked and hourly rates. Rising unemployment and falling income can act directly, by constraining the abilities of families to afford mortgages and market rents, or indirectly, with emotional distress leading to mental health problems, domestic violence and, ultimately, family breakdown.^11,17,18^ Job loss may particularly increase risk of homelessness where welfare safety nets are weak.^11^

In England, there is highly polarized debate about what is causing the recent rise in homelessness. One group of commentators, mostly NGOs and public health charities, express concern that budgetary cuts to funding for homelessness prevention, housing benefits, and social services are a major factor; for these play critical buffering roles during times of hardship experienced by impoverished households.^16,19–21^ Across England, local government spending, including spending on homelessness prevention, has been reduced in real terms by 27% over 2010–15.^22^ Another group, composed largely of the politicians responsible for the austerity measures, have suggested the cuts would not impact on homelessness risk. For example, in 2012, then Deputy Prime Minister Nick Clegg said that the cuts were not ‘some sort of punitive programme of mass homelessness’.^23^ Kris Hopkins, Housing Minister in 2014, dismissed research linking welfare reforms to increasing homelessness,^16^ stating the government had increased funding to prevent homelessness and help people sleeping rough.^24^

As argued by Schrecker and Milne in a recent *Journal of Public Health* editorial*,* these debates have particular relevance to public health because there is a marked lack of evidence on the impact of public spending cuts on health.^15^ Relatively little is known about which, if any, specific social protection programmes can prevent risks of homelessness, especially during times of economic crisis.^11^ This debate has wider relevance in Europe, as Spain, Portugal, Greece and other nations are also experiencing substantial reductions in spending on social programmes.^25^ Thus, there is a critical need for empirical investigation of the impact of recession and public investment on homelessness.

In this study we exploit the marked variation in homelessness claim rates across 323 local authorities to evaluate the structural economic determinants of homelessness in England during the years prior to the recession (2004–07), during the economic downturn (2007–10), and the subsequent implementation of fiscal austerity measures (2010–12). We use data on statutory homelessness, which captures those who seek emergency homelessness assistance. While it is not a comprehensive measure of homelessness, as it excludes those who do not seek help, including many rough sleepers, it is the only source of comparable data over time at local authority level. We explore the role of the two major factors outlined above, economic downturns and loss of social safety nets, with a particular focus on cuts to housing services and social care.

## Methods

### Data and variables

We collected homelessness, unemployment and government expenditure data for 326 lower tier local authorities in England. All data covered fiscal years from 2004/05 to 2012/13, subsequently denoted by the base year, and data were harmonized to account for boundary shifts in 2009 (see Supplementary data, Appendix B for more details). The final analytical sample included 323 local authorities for a total of 2907 local authority-years.

We use data from local authorities on the number of households making claims to their local authority for homelessness assistance, known as statutory homelessness claims. These data can be used as a proxy measure of emerging need as they capture the number of households who are newly or imminently unable to secure their own shelter (see Supplementary data, Appendix B for eligibility criteria and legislation).^14^ Local authorities report these statistics on an annual basis to the Department of Communities and Local Government. The total number of claims reflects households who meet criteria for receiving statutory homelessness assistance (accepted claims), and those who did not (unaccepted claims). Data on accepted and unaccepted claims were missing or incomplete for 77 local authority-years (2.7% of observations), and in an additional 20 local authority-years only accepted claims were reported, precluding calculation of total claims.

To test the effects of the severity of recessions, we included data on unemployment and Gross Value Added per capita, a commonly used measure of economic activity. Unemployment data come from the official database of labour market statistics for the UK (Nomis) and are annual working-age unemployment rates estimated from the Annual Population Survey.^26^ Gross Value Added per capita is the total production of goods and services by residents and corporations in 99 NUTS (Nomenclature of Territorial Units for Statistics) Level 3 regions in England. Since Gross Value Added per capita values were right skewed, they were logged in all analyses and coefficients were presented as semi-elasticities to facilitate interpretation.

We assessed welfare expenditure by central government and local authorities. We obtained data on expenditure for centrally allocated welfare benefits for each local authority from the UK Department of Work and Pensions.^27^ Local authorities' expenditures support the provision of housing, social care, and planning and development, among others.^28^ We obtained these data from the UK Department of Communities and Local Government^28^ and summed expenditure across these categories, except education (because spending is not comparable between years).^29^ In shire counties, upper tier councils determine spending on social care, transportation, libraries and strategic planning; to calculate expenditures on these at the lower district level we scaled expenditure to districts according to share of the population in their associated upper tier councils. Supplementary data, Appendix B further details the structure of welfare expenditure in England and the measures included in our analysis. All expenditure data were adjusted for inflation and are presented in constant 2012 British pounds on a per capita basis.

We further disaggregated welfare expenditure into spending on social care and housing, separated because these areas directly impact upon populations at risk of homelessness. Importantly, spending on housing encompasses local authority investment in homelessness prevention. Because spending on housing services also includes the cost of providing accommodation and welfare for homeless households, it was necessary to remove this from spending in this category. We did so by obtaining detailed data from the Department of Communities and Local Government on housing services expenditure and recalculating housing expenditure after removing spending on homelessness accommodation, administration and welfare.

### Statistical analysis

Our statistical models aim to account for the recent rise in homelessness and thus focus on marked short-term annual changes in homelessness within local authorities. However we note that long-term changes may have alternative determinants. Thus our cross-local authority fixed effects models are as follows to test the impact of economic factors that fluctuate annually:

(1) $$\begin{array}{ll} \Delta \mathrm{Homelessness}\;\;\mathrm{Claim}s_{it} & =\beta_{0}\\ & +\beta_{1}\Delta \mathrm{Log}\;\;\mathrm{Gross}\;\;\mathrm{Value}\;\;\mathrm{Adde}d_{it}\\ & +\beta_{2}\Delta \;\mathrm{Unemploymen}t_{it}\\ & +\beta_{3}\%\Delta \;\mathrm{Local}\;\;\mathrm{Expenditur}e_{it}\\ & +\beta_{4}\%\Delta \;\mathrm{Central}\;\;\mathrm{Expenditur}e_{it}\\ & +\mu_{i}+\epsilon_{it} \end{array}$$

Here *i* is local authority and *t* is year. Δ denotes the annual change. Gross Value Added is the annual percentage change and coefficients are reported as semi-elasticities; Unemployment is the annual percentage point change in rates; Local expenditure is percentage changes in local authority spending per capita; and Central expenditure is the percentage changes central government spending on benefits in each local authority. *μ* are local authority fixed effects. We use nested models to first examine the effects of economic recession, followed by the addition of changes in welfare expenditure.

Next, to identify which specific budgetary headings are most strongly linked with homelessness trends, we evaluate the main components of welfare expenditure that plausibly affect populations at risk of homelessness. While much concern about spending cuts and welfare reforms have focused on changes to housing benefit and homelessness prevention spending, we examine the full range of income supports which could impact households' ability to afford housing, and spending on social care services as well, since these impact groups vulnerable to homelessness, including low income pensioners, people with disabilities, and people who are unemployed.

Because use of per cent change values can skew the distribution if there are areas that had very low baseline values, we excluded observations where absolute percentage differences in spending exceeded 500% (*n* = 14 local authority-year observations) because these changes were implausible with respect to their impact on outcome variable of interest. Excluding these observations did not substantively change our findings but improved model fit. Robust standard errors were calculated adjusting for heteroskedasticity and clustering of local authorities. All models were estimated using STATA v13.

## Results

### Trends in homelessness rates across England

Between 2004 and 2009, the mean homelessness rate in local authorities declined from 10.4 per 1000 households to 3.5 per 1000 households (Fig. 1). Subsequently, homelessness claims began rising, increasing, on average, by 0.33 claims per 1000 households between 2010 and 2011 to a rate of 4.1 per 1000. Rates remained relatively stable through 2012. As shown in Fig. 2, these rises varied across local authorities. Approximately one in five local authorities experienced a rise of two or more claims per 1000 households over this period. In Islington, Luton, and, Canterbury, for example, appeals for homelessness support jumped from about 4 claims per 1000 households in 2009 to 13 or more claims per 1000 households in 2012. In contrast, one-third of local authorities experienced an overall decline in the number of claims.

At the same time that homelessness claims were decreasing overall, total per capita spending on welfare services by local authorities rose from 2004 to 2009, but thereafter fell annually by, on average, 5.2% (Supplementary data, Figure SA1). Per capita spending by central government on welfare benefits remained fairly constant until 2008, but then rose, reflecting their role as automatic stabilizers during the recessionary period. From 2009, per capita spending began to fall.

### Macroeconomic determinants of homelessness rates

First, we examined the relationships between unemployment rates and Gross Value Added and homelessness claims (Table 1). There was no effect of changes in unemployment rate. In unadjusted models, each 10% fall in Gross Value Added per capita was associated with an increase of 0.29 total homelessness claims per 1000 households (95% CI: 0.01–0.58). After adjusting for the effects of government spending, this relationship was stronger; we found that each 10% decline in Gross Value Added per capita was linked with a 0.48 rise in total homelessness claims (95% CI: 0.10–0.80).

**Table 1:** Association of economic and welfare expenditure changes with changes in homelessness claim rates across 323 local authorities in England, 2004–12

	*Change in homelessness claim rate (per 1000 households)*	
	*(1)*	*(2)*
Percentage change in Gross Value Added per capita	−0.029* (0.015)	−0.045* (0.018)
Change in unemployment rate	−0.024 (0.019)	−0.0044 (0.021)
Percentage change in local authority expenditure	—	−0.083*** (0.011)
Percentage change in central government expenditure	—	−0.016 (0.020)
Number of local authority-years	2167	2167
*R*^2^	0.0030	0.0470

*Notes:* Standard errors estimated using fixed effects in parentheses clustered by local authority to reflect non-independence of sampling. Number of local authorities = 323.

*P < 0.05, ***P < 0.001.

Adding welfare expenditure from central government and local authorities to these models (Model 2 of Table 1), showed that each 10% cut in local authority spending was significantly associated with a 0.83 (95% CI: 0.62–1.03) increase in the total homelessness claim rate per 1000 households.

### Testing adverse effects of budget cuts

Table 2 shows the results of the cross-local authority statistical models, adjusted for change in Gross Value Added per capita and unemployment rates. First, we disaggregated housing services and social care from other types of local authority spending. A 10% decrease in spending on housing, including investment in homelessness prevention, was associated with a rise of 0.03 (95% CI: 0.01–0.04) in total homelessness claims per 1000 households, respectively. Changes in social care spending were significantly related to total claim rates, such that a 10% decrease in spending was associated with increases of 0.80 (95% CI: 0.54–1.06), 0.27 (95% CI: 0.08–0.47) and 0.40 (95% CI: 0.24–0.57) in total claims. Spending on other elements of local authority services was also associated with total claim rates.

**Table 2:** Association of welfare services and benefit expenditure changes with changes in homelessness claim rates across 323 local authorities in England, 2004–12

	*Change in homelessness claim rate (per 1000 households)*		
	*(1)*	*(2)*	*(3)*
Percentage change in:			
Local authority expenditure			
Housing services	−0.0028** (0.00090)	—	−0.0013 (0.00088)
Social care	−0.080*** (0.014)	—	−0.046*** (0.013)
Other services	−0.025*** (0.0054)	—	−0.012* (0.0056)
Central government expenditure			
Employment and support allowance^a^	—	0.11** (0.035)	0.093** (0.034)
Job seekers allowance	—	−0.00086 (0.0035)	0.0021 (0.0036)
Disability living allowance	—	−0.076* (0.037)	−0.056 (0.038)
Housing benefit	—	0.016 (0.021)	0.013 (0.021)
Discretionary housing payments	—	−0.0017* (0.00067)	−0.0016* (0.00069)
Council tax benefit	—	−0.015 (0.024)	−0.013 (0.024)
Pension credit	—	−0.12*** (0.021)	−0.096*** (0.022)
Pension-age disability support^b^	—	−0.092** (0.034)	−0.085** (0.033)
Number of local authority-years	2167	2161	2161
*R*^2^	0.0529	0.0960	0.1094

*Notes:* All models adjusted for change in Gross Added Value and unemployment but estimates not shown. Robust standard errors in parentheses clustered by local authority to reflect non-independence of sampling. Number of local authorities = 323.

^a^Includes employment and support allowance, incapacity benefit, disability-related income support, and severe disablement allowance for working-age claimants.

^b^Includes attendance allowance and severe disability payments for pension-age claimants.

*P < 0.05, **P < 0.01, ***P < 0.001.

Second, we examined major areas of central welfare spending, as shown in Table 2. The most important areas were investment in benefits to support the living costs of individuals living with disability and for low income seniors. We observed that each 10% decrease in spending on Pension Credits was associated with a 1.16 per 1000 household (95% CI: 0.73–1.57) rise in the total number of homelessness claims across local authorities. Decreasing spending on discretionary housing payments was also significantly associated with rising total homelessness claims. Spending on Job Seekers Allowance and Housing Benefits had no effect. Employment and Support Allowance expenditure was positively associated with increases in homelessness, which was plausible, reflecting a role as ‘automatic stabilizers’, such that spending automatically rises when hardship increases. As shown in Table 2, when we incorporated both local authority and central government spending in a combined model, we found that effect sizes of the former were attenuated reflecting a larger number of covariates, but that the observed patterns did not qualitatively differ.

Upon observing that social programmes that affect older persons were associated with rising homelessness, we used the available national level data for England to examine trends in accepted homelessness claims by age bands (Supplementary data, Figure SA2). As shown in the figure, trends in the annual change in homelessness were generally consistent across age categories. However, homelessness applicants in the 65–74 group and 75 and over group experienced the largest per cent rises in 2010/11, and the 65–74 age group experienced a pronounced increase in 2012/13, which coincides with the period of reductions in pension credits which occurred in 2011 and 2012.

### Robustness checks

We performed several tests of our model's specification and sample. We replicated the fixed effects models adding an annual time trend to account for pre-existing downward linear trend evident before 2010 across local authorities. Results were attenuated but remained significant for the change in local authority spending in relation to total homelessness claims (Supplementary data, Table SA2). In models examining disaggregated categories of expenditure, social care spending remained statistically significant but spending on housing was attenuated to non-significance, and for central authority expenditure the main factors remained discretionary housing payments and pension credit spending (Supplementary data, Table SA3). We also replicated our study using unaccepted and accepted claims as outcomes separately, finding similar patterns across both (Supplementary data, Table SA4). Given potential measurement error in labour force surveys of unemployment rates, we also test the associations of employment rates and economic inactivity rate. These measures had no detectable effect (Supplementary data, Table SA5).

Additionally, we considered other variables that have been found to associate with rates of homelessness across local authorities in England. Specifically, we examined changes in the density of benefit claimants at working and pension ages, internal and international immigration into local authorities, and the housing market. With respect to the latter, we incorporated data on housing affordability for low income households (i.e. the ratio of the lower quartile housing prices to lower quartile earnings), social housing wait lists, and local authority dwelling stock (Supplementary data, Table SA6). Incorporating these measures did not change the observed association between homelessness and local authority spending, but attenuated the relationship with GVA. Increasing density of beneficiaries of pension credit (welfare support for low income pensioners) was associated with declining rates of homelessness; the converse of this could suggest that reduced eligibility and coverage of pension credit, which occurred in 2011, could be contributing to rising homelessness, and may also underlie the inverse association observed between spending in this area and homelessness. There was a positive association between social housing wait lists and rising homelessness, which suggests that, in areas of rising pressure on social housing, there are more households making homelessness applications. Other housing market and immigration variables were not associated with changes in the rate of homelessness applications.

Lastly, using so-called ‘Granger causality’ tests,^30^ we evaluated the possibility that increasing homelessness claims in the previous year could drive changes in spending in following year. To do so we evaluated which came first, homelessness or spending rises. Our model included the change in homelessness claim rate in the year prior, finding that it was not significant, but that the local authority spending change in the year prior was inversely associated with homelessness claims as well as the contemporaneous spending change we observed (Supplementary data, Table SA7). Rising homelessness rates in previous year were negatively associated with changes in spending in the following year (Supplementary data, Table SA8), which could reflect that when there is rising homelessness in a preceding year, there is more demand for local authority resources for homelessness provision (not included in our spending variable), which results in funds being diverted from other areas of spending. Together, this evidence supports the notion that budget cuts temporally preceded homelessness rises (i.e. budget cuts ‘Granger cause’ homelessness).

## Discussion

### Main findings of this study

Our cross-local area statistical models establish that variation in annual changes in homelessness claim rates across local authorities in England is associated with structural economic changes. We found that reductions in spending on social welfare by local authorities and central government were strongly associated with increased homelessness. At the local authority level, spending on social care and housing support programmes were major contributing factors. At the central government level, spending on pension support, disability allowances for pensioners and discretionary housing payments were significant factors.

### What is already known on this topic

Housing is a critical determinant of health; exposure to homelessness is associated with declines in mental health and higher risk of chronic and infectious diseases and physical harm.^2^ Prior studies of structural economic determinants of homelessness have largely been conducted in the USA, finding that high unemployment, rent burdens and low social security are risk factors.^12,13^ Relatively less work has been done in the UK, although cross-sectional research revealed higher rates in areas that were more deprived, had high housing prices, and greater proportions of high-risk groups, such as young adults and migrants.^14^ Additionally there was evidence that local authority actions to prevent homelessness corresponded to lower statutory homelessness rates.

Since 2010 the UK has experienced a marked rise in homelessness appeals, with wide variations across local authorities and over time. The reasons are not well understood but have been speculated as pertaining to austere welfare reforms, especially to homelessness prevention programmes, and recent increases in economic hardship.^16^

### What this study adds

There is ongoing debate about how welfare reforms and spending changes made since 2010 have impacted the health and vulnerability of people in England.^17,21^ To our knowledge for the first time this study demonstrates that budget reductions are a significant contributing factor to rises in homelessness.

Further, we disaggregate alternative categories of welfare spending to highlight those areas of expenditure most likely to pose threats of homelessness if reduced. These include spending on housing and discretionary housing payments, but also areas of welfare reforms that have not undergone much scrutiny, specifically, spending on social care, which affects vulnerable adult populations and income support for low income seniors. While many programmes are not ostensibly aimed at reducing homelessness, we find that they can have as great, if not greater, impact on risk to vulnerable groups.

Taken together, our findings highlight the important areas of investment in welfare to prevent homelessness. They also suggest that austerity measures in the UK could be undermining progress on reductions in homelessness and leading to a new generation of homeless families and individuals. Importantly, in light of recent announcements of welfare benefit freezes, reductions to welfare support for families, and projected reduction of funds for local authorities in the Summer 2015 budget,^31^ these findings suggest that local authorities and homelessness charities need to be prepared for rising housing need in their populations.

### Limitations of this study

First, the data used are at the area level, creating potential for ecological fallacies. Unfortunately, individual level data on homeless populations are unavailable, reflecting the difficulty tracking individual cases over time and their socio-demographic characteristics. As shown, our models explained relatively little of the annual fluctuation in homelessness rates in local authorities. While including fixed effects and modelling changes means that we were able to correct for static differences between local authorities with respect to surveillance variation, we were unable to account for unobserved procedural changes that occurred within local authorities over time.^16,32^

Second, since 2011, local authorities have been able to absolve their homelessness duty by securing accommodation for homeless households in the private rented sector. This recent change may mean that fewer households are applying for homelessness assistance because the support offered is not appropriate. Other indicators of housing precariousness suggest that rates of application for homelessness assistance are not fully capturing rising housing need in England.^16^ There is also some evidence that local authorities have changed the ways they process homelessness applications, sometimes acting to divert individuals from making applications.^32^ Thus data on appeals for homelessness assistance likely understate the full burden of homelessness and do not capture the experiences of the most vulnerable populations. Those discouraged from making a claim because they do not meet eligibility criteria or using other coping strategies to manage the threat or experience of losing their housing (e.g. moving in with friends or family) were not captured in the data, nor were single individuals living in shelters or sleeping rough.^10^ This highlights the need for improving surveillance of homelessness to capture those who do not interact with the housing support system.

Third, we used financial data to compare welfare services and benefits across local authorities and over time. However, what matters is not just the magnitude of spending but its effectiveness. Structural changes to welfare service delivery could be financially neutral but mitigate or exacerbate homelessness risks. Future research is needed to understand how UK welfare reforms implemented after 2012 impact on homelessness risk, in particular the introduction of benefit caps and the under-occupancy charge (also known as the bedroom tax) for households receiving housing benefit. Lastly, there is widespread interest in developing a National Homelessness Monitoring System across Europe to facilitate cross-national comparisons and better understanding of the root social and economic drivers of homelessness.^33^ The ability to generalize these findings to other country contexts is currently unknown and likely limited by the unique statutory homelessness system in England.

## Supplementary data

Supplementary data are available at the *Journal of Public Health* online.

## Funding

D.S. and R.L. are funded by a Wellcome Trust Investigator Award.

## Supplementary Material

Supplementary Data
