# The impact of economic downturns and budget cuts on homelessness claim rates across 323 local authorities in England, 2004–12

**DOI:** 10.1093/pubmed/fdx083

**Published:** 2017-07-13

**Authors:** Rachel Loopstra, Aaron Reeves, Ben Barr, David Taylor-Robinson, Martin McKee, David Stuckler

*J Public Health (Oxf)* 2016; 38 (3): 417–425. doi: 10.1093/pubmed/fdv126

The funding information should have included the following statement:

DS is funded by ERC HRES 313590 and a Wellcome Trust Investigator Award.

The online article has been corrected.

