# Supplementary material for: The impact of economic downturns and budget cuts on homelessness claim rates across 323 local authorities in England, 2004–12
Source: J Public Health (Oxf). 2016 Oct 17;38(3):417–25. doi: 10.1093/pubmed/fdv126 (PMC5072163; doi:10.1093/pubmed/fdv126)
Supplement: Supplementary Data [file supp_38_3_417__index.html]

The impact of economic downturns and budget cuts on homelessness claim rates across 323 local authorities in England, 2004–12 — The impact of economic downturns and budget cuts on homelessness claim rates across 323 local authorities in England, 2004–12 — Supplementary Data 

# The impact of economic downturns and budget cuts on homelessness claim rates across 323 local authorities in England, 2004–12

## Supplementary Data

Supplementary Data

- Supplementary Appendix A - docx file
- Supplementary Appendix B - docx file
