# Supplementary material for: The impact of economic downturns and budget cuts on homelessness claim rates across 323 local authorities in England, 2004–12
Source: J Public Health (Oxf). 2016 Oct 17;38(3):417–25. doi: 10.1093/pubmed/fdv126 (PMC5072163; doi:10.1093/pubmed/fdv126)
Supplement: Supplementary Data [file supp_fdv126_fdv126supp_AppendixA.docx]

**Web Appendix A: Additional Tables and Figures**

Web Figure A1. Trends in central government and local authority welfare expenditure in England, 2004-2012.

Web Figure A2 Changes in homeless households in priority need accepted by local authorities by age of applicant, England 2006/07 to 2012/13.

Web Table A1. Descriptive statistics for 323 English local authorities from 2004-2012.

Web Table A2. Association of Welfare Services and Benefit Expenditure Changes with Homelessness Rates in England with adjustment for linear time trends, 323 Local Authorities, 2004-2012.

Web Table A3. Association of Welfare Services and Benefit Expenditure Changes with Homelessness Rates in England with adjustment for linear time trend, 323 Local Authorities, 2004-2012.

Web Table A4. Association of Economic and Welfare Expenditure Changes with Total, Accepted, and Unaccepted Homelessness Claim Rates in England, 323 Local Authorities, 2004-2012.

Web Table A5. Association of Welfare Services and Benefit Expenditure Changes with Homelessness Rates in England with adjustment for employment rate and economic inactivity rate, 323 Local Authorities, 2004-2012.

Web Table A6. Association of Welfare Services and Benefit Expenditure Changes with Homelessness Rates in England with adjustment for changes in benefit claimant density, international and internal immigration, housing market variables, and linear time trend, 323 Local Authorities, 2004-2012.

Web Table A7. Association of Welfare Services and Benefit Expenditure Changes with Homelessness Rates in England with adjustment for lagged rates of changes in homelessness claims, and linear time trend, 323 Local Authorities, 2004-2012.

Web Table A8. Association of Previous Year Homelessness Rates with Local Authority Expenditure Changes in England and linear time trend, 323 Local Authorities, 2004-2012.

Web Figure A1. Trends in central government and local authority welfare expenditure in England, 2004-2012.

*Notes:* Authors’ calculations from [^22^](#_ENREF_22) [^26^](#_ENREF_26).

Web Figure A2 Annual percent change in accepted homeless claims in priority need by local authorities by age of applicant, England 2006/07 to 2012/13.

Source: Department of Communities and Local Government.

Web Table A1. Descriptive statistics for 323 English local authorities from 2004-2012.

| Variable | Number of Observations | Mean | SD | Min | Max |
| --- | --- | --- | --- | --- | --- |
| *Homelessness Claims (per 1000 households)* |  |  |  |  |  |
| Total Claims | 2797 | 5.54 | 4.93 | 0.08 | 40.16 |
| Accepted Claims | 2817 | 2.58 | 2.12 | 0.00 | 15.13 |
| Unaccepted Claims | 2797 | 2.96 | 3.45 | 0.00 | 34.94 |
| *Change in Homelessness Claims (per 1000 households)* |  |  |  |  |  |
| Total Claims | 2412 | -0.77 | 2.34 | -16.61 | 19.67 |
| Accepted Claims | 2441 | -0.35 | 1.09 | -9.91 | 10.41 |
| Unaccepted Claims | 2412 | -0.42 | 1.69 | -15.35 | 9.27 |
| *Per Capita Local Authority Welfare Expenditure (2012 £)* |  |  |  |  |  |
| Total Spending on Services | 2893 | 792.38 | 200.51 | 531.32 | 1839.39 |
| Housing | 2893 | 36.46 | 29.48 | -29.69 | 263.04 |
| Social Care | 2893 | 392.04 | 74.43 | 173.60 | 794.49 |
| All Other Services | 2893 | 363.88 | 123.50 | 192.16 | 995.30 |
| *Percentage Change in Local Authority Welfare Spending* |  |  |  |  |  |
| Total Spending on Services | 2570 | -0.75 | 6.19 | -26.69 | 46.87 |
| Housing | 2570 | 3.15 | 56.59 | -393.99 | 447.35 |
| Social Care | 2570 | 0.63 | 6.57 | -33.98 | 180.22 |
| All Other Services | 2570 | -1.52 | 11.06 | -48.00 | 124.77 |
| *Per Capita Central Government Welfare Expenditure (2012 £)* |  |  |  |  |  |
| Total Benefits | 2893 | 1143.84 | 367.54 | 445.03 | 2490.53 |
| Employment and Support & Related Benefits | 2893 | 199.36 | 81.89 | 66.29 | 539.19 |
| Job Seekers Allowance | 2893 | 53.37 | 30.77 | 9.10 | 205.93 |
| Disability Living Allowance | 2893 | 170.40 | 63.70 | 61.13 | 488.79 |
| Housing Benefit | 2893 | 293.66 | 168.05 | 74.17 | 1347.73 |
| Discretionary Housing Payments | 2893 | 0.36 | 0.47 | 0.00 | 16.21 |
| Council Tax Benefit | 2893 | 74.17 | 23.63 | 26.76 | 189.04 |
| Pension Credit | 2893 | 121.91 | 37.98 | 40.68 | 257.52 |
| Pension-age Disability Support^2^ | 2893 | 86.44 | 22.84 | 35.34 | 170.13 |
| Winter Fuel Allowance | 2893 | 47.04 | 13.02 | 14.83 | 113.58 |
| Carer's Allowance | 2893 | 23.42 | 8.74 | 8.49 | 67.02 |
| Income Support (Single Parents, Carer, Other) | 2893 | 60.90 | 37.81 | 11.81 | 300.56 |
| Bereavement Benefits | 2893 | 12.80 | 3.67 | 3.36 | 24.16 |
| *Percentage Change in Central Government Welfare Expenditure* |  |  |  |  |  |
| Total Benefits | 2570 | 1.51 | 3.39 | -7.94 | 14.95 |
| Employment and Support & Related Benefits | 2570 | 2.38 | 3.10 | -6.70 | 14.10 |
| Housing Benefit | 2570 | -9.40 | 4.39 | -23.87 | 7.31 |
| Discretionary Housing Payments | 2570 | -1.33 | 2.88 | -10.91 | 7.48 |
| Council Tax Benefit | 2570 | -8.10 | 4.95 | -23.38 | 25.60 |
| Disability Living Allowance | 2570 | 4.48 | 3.70 | -8.45 | 16.52 |
| Job Seekers Allowance | 2570 | 1.67 | 4.21 | -11.37 | 18.04 |
| Pension Credit | 2562 | 24.90 | 78.82 | -100.00 | 1083.64 |
| Winter Fuel Allowance | 2570 | 3.91 | 2.05 | -3.52 | 10.31 |
| Pension-age Disability (Attendance Allowance, Severe Disability Pension Age) | 2570 | 5.23 | 4.70 | -12.32 | 27.13 |
| Carer's Allowance | 2570 | 11.29 | 26.30 | -30.10 | 117.15 |
| Income Support (Single Parents, Carer, Other) | 2570 | 0.20 | 5.04 | -13.19 | 31.31 |
| Bereavement benefits | 2570 | -2.51 | 20.18 | -40.58 | 29.23 |
| *Indicators of Recession* |  |  |  |  |  |
| GVA per Capita, £ | 2893 | 21754 | 13771 | 11296 | 132904 |
| Percentage Change in Gross Value Added per Capita | 2570 | -0.35 | 3.77 | -12.65 | 15.72 |
| Unemployment Rate | 2738 | 6.20 | 2.85 | 1.00 | 18.80 |
| Change in Unemployment Rate | 2316 | 0.41 | 2.34 | -11.50 | 15.10 |

*Notes:* Sources of data from UK Department of Work and Pensions and Communities and Local Government, NOMIS Labour Market Statistics, and Office of National Statistics.

Web Table A2. Association of Economic and Welfare Expenditure Changes with Homelessness Rates in England, 323 Local Authorities with adjustment for linear time trend, 2004-2012

|  | Change in Homelessness Claim Rate  (per 1,000 Households) | |
| --- | --- | --- |
|  | (1) | (2) |
| Percentage Change in Gross Value Added per Capita | 0.041^**^ | 0.040^*^ |
|  | (0.015) | (0.019) |
| Change in Unemployment Rate | 0.0094 | 0.0093 |
|  | (0.019) | (0.020) |
| Percentage Change in Local Authority Expenditure | — | -0.030^**^ |
|  |  | (0.010) |
| Percentage Change in Central Government Expenditure | — | 0.020 |
|  |  | (0.020) |
| Number of Local Authority-Years | 2167 | 2167 |
| *R*^2^ | 0.1350 | 0.1401 |

*Notes:* Robust standard errors in parentheses clustered by Local Authority to reflect non-independence of sampling. Number of local authorities = 323.

^*^ *p* < 0.05, ^**^ *p* < 0.01, ^***^ *p* < 0.001

Web Table A3. Association of Welfare Services and Benefit Expenditure Changes with Homelessness Rates in England with adjustment for linear time trend, 323 Local Authorities, 2004-2012.

|  | Change in Homelessness Claim Rate  (per 1,000 Households) | | |
| --- | --- | --- | --- |
| *Percentage Change in:* | (1) | (2) | (3) |
| *Local Authority Expenditure* |  |  |  |
| Housing Services | -0.0011 | — | -0.0010 |
|  | (0.00083) |  | (0.00085) |
| Social Care | -0.034^**^ | — | -0.036^**^ |
|  | (0.012) |  | (0.012) |
| Other Services | -0.0051 | — | -0.0034 |
|  | (0.0052) |  | (0.0056) |
| *Central Government Expenditure* |  |  |  |
| Employment and Support Allowance^1^ | — | -0.022 | -0.027 |
|  |  | (0.035) | (0.036) |
| Job Seekers Allowance | — | -0.0026 | -0.00082 |
|  |  | (0.0034) | (0.0035) |
| Disability Living Allowance | — | 0.0011 | 0.013 |
|  |  | (0.039) | (0.039) |
| Housing Benefit | — | -0.029 | -0.028 |
|  |  | (0.022) | (0.022) |
| Discretionary Housing Payments | — | -0.0016^*^ | -0.0015^*^ |
|  |  | (0.00068) | (0.00070) |
| Council Tax Benefit | — | 0.018 | 0.020 |
|  |  | (0.024) | (0.024) |
| Pension Credit | — | 0.096^*^ | 0.096^*^ |
|  |  | (0.043) | (0.041) |
| Pension-age Disability Support^2^ | — | -0.035 | -0.035 |
|  |  | (0.032) | (0.032) |
| Observations | 2167 | 2161 | 2161 |
| *R*^2^ | 0.14 | 0.15 | 0.16 |

*Notes:* All models adjusted for changes in Gross Added Value, percentage point changes in unemployment rates, and a linear time trend. Robust standard errors in parentheses clustered by Local Authority to reflect non-independence of sampling. Number of local authorities = 323.

^1^ Includes Employment and Support Allowance, Incapacity Benefit, Disability-related Income Support, and Severe Disablement Allowance for working-age claimants. ^2^ Includes Attendance Allowance and Severe Disability Payments for pension-age claimants.

^*^ *p* < 0.05, ^**^ *p* < 0.01, ^***^ *p* < 0.001

Web Table A4. Association of Economic and Welfare Expenditure Changes with Total, Accepted, and Unaccepted Homelessness Claim Rates in England, 323 Local Authorities, 2004-2012.

|  | Change in Homelessness Rates per 1,000 Households | | | | | |
| --- | --- | --- | --- | --- | --- | --- |
|  | Total Homelessness Claims | | Accepted Homelessness Claims | | Unaccepted Homelessness Claims | |
|  | (1) | (2) | (3) | (4) | (5) | (6) |
| Percentage Change in Gross Value Added per Capita | -0.029^*^ | -0.045^*^ | -0.0049 | -0.018^*^ | -0.025^*^ | -0.028^*^ |
|  | (0.015) | (0.018) | (0.0075) | (0.0087) | (0.011) | (0.012) |
| Change in Unemployment Rate | -0.024 | -0.0044 | -0.0093 | 0.0028 | -0.014 | -0.0062 |
|  | (0.019) | (0.021) | (0.011) | (0.012) | (0.013) | (0.014) |
| Percentage Change in Local Authority Expenditure | — | -0.083^***^ | — | -0.039^***^ | — | -0.044^***^ |
|  |  | (0.011) |  | (0.0049) |  | (0.0075) |
| Percentage Change in Central Government Expenditure | — | -0.016 | — | -0.022^*^ | — | 0.0049 |
|  |  | (0.020) |  | (0.010) |  | (0.014) |
| Number of Local Authority-Years | 2167 | 2167 | 2194 | 2194 | 2167 | 2167 |
| *R*^2^ | 0.0030 | 0.0470 | 0.0011 | 0.0521 | 0.0033 | 0.0260 |

*Notes:* Standard errors estimated using fixed effects in parentheses clustered by Local Authority to reflect non-independence of sampling. Number of local authorities = 323.

^*^ *p* < 0.05, ^**^ *p* < 0.01, ^***^ *p* < 0.001

Web Table A5. Association of Welfare Services and Benefit Expenditure Changes with Homelessness Rates in England with adjustment for employment rate and economic inactivity rate, 323 Local Authorities, 2004-2012.

|  | | Change in Homelessness Claim Rate  (per 1,000 Households) | | | |
| --- | --- | --- | --- | --- | --- |
|  | (1) | | (2) | (3) | (4) |
| Percentage Change in Gross Value Added per Capita | -0.032^*^  (0.013) | | -0.044^**^  (0.015) | -0.032^*^  (0.013) | -0.044^**^  (0.015) |
| Change in Employment Rate | 0.007  (0.011) | | 0.0001  (0.012) |  |  |
| Change in Economic Inactivity Rate |  | |  | 0.0026  (0.012) | 0.0034  (0.012) |
| Percentage Change in Local Authority Expenditure |  | | -0.082^***^  (0.009) |  | -0.082^***^  (0.009) |
| Percentage Change in Central Government Expenditure |  | | -0.009  (0.017) |  | -0.009  (0.017) |
| Number of Local Authority-Years | 2412 | | 2412 | 2412 | 2412 |
| *R*^2^ | 0.003 | | 0.054 | 0.003 | 0.054 |

*Notes:* Standard errors estimated using fixed effects in parentheses clustered by Local Authority to reflect non-independence of sampling. Number of local authorities = 323.

^*^ *p* < 0.05, ^**^ *p* < 0.01, ^***^ *p* < 0.001

Web Table A6. Association of Welfare Services and Benefit Expenditure Changes with Homelessness Rates in England with adjustment for changes in benefit claimant density, international and internal immigration, housing market variables, and linear time trend, 323 Local Authorities, 2004-2012.

|  | Change in Homelessness Claim Rate  (per 1,000 Households) | | | | |
| --- | --- | --- | --- | --- | --- |
|  | (1) | (2) | (3) | (4) | (5) |
| Percentage Change in Gross Value Added per Capita | -0.029^*^ | -0.045^**^ | 0.012 | 0.012 | 0.015 |
|  | (0.014) | (0.016) | (0.017) | (0.017) | (0.018) |
| Change in Unemployment Rate | -0.024 | -0.0044 | 0.012 | 0.012 | 0.018 |
|  | (0.018) | (0.019) | (0.020) | (0.019) | (0.020) |
| Percentage Change in Local Authority Expenditure |  | -0.083^***^ | -0.050^***^ | -0.050^***^ | -0.049^***^ |
|  |  | (0.0098) | (0.0099) | (0.0100) | (0.010) |
| Percentage Change in Central Government Expenditure |  | -0.016 | 0.035 | 0.034 | 0.037 |
|  |  | (0.019) | (0.025) | (0.025) | (0.025) |
| Per 1% increase in DWP working-age benefit recipients |  |  | -0.048 | -0.058 | -0.074 |
|  |  |  | (0.16) | (0.17) | (0.18) |
| Per 1% increase in pension credit beneficiaries |  |  | -0.58^***^ | -0.58^***^ | -0.51^***^ |
|  |  |  | (0.058) | (0.068) | (0.073) |
| Per 1 immigrant increase per 1000 people from abroad |  |  |  | 0.0019 | 0.0053 |
|  |  |  |  | (0.027) | (0.027) |
| Per 1 person increase per 1000 people from other local authority area |  |  |  | -0.0060 | -0.0011 |
|  |  |  |  | (0.015) | (0.015) |
| Change in Dwelling Stock |  |  |  |  | -0.00045 |
|  |  |  |  |  | (0.00025) |
| Change in Number on Social Housing Waiting List |  |  |  |  | 0.00075^*^ |
|  |  |  |  |  | (0.00030) |
| Change in Ratio of Low Income Housing Price to Earnings |  |  |  |  | -0.00046 |
|  |  |  |  |  | (0.076) |
| Number of Local Authority-Years | 2167 | 2167 | 2167 | 2167 | 2140 |
| *R*^2^ | 0.002 | 0.056 | 0.128 | 0.128 | 0.135 |

*Notes:* Standard errors estimated using fixed effects in parentheses clustered by Local Authority to reflect non-independence of sampling. Number of local authorities = 323.

^*^ *p* < 0.05, ^**^ *p* < 0.01, ^***^ *p* < 0.001

Web Table A7. Association of Welfare Services and Benefit Expenditure Changes with Homelessness Rates in England with adjustment for lagged rates of changes in homelessness claims, 323 Local Authorities, 2004-2012.

|  | | Change in Homelessness Claim Rate (per 1,000 Households) |
| --- | --- | --- |
|  | (1) | |
| Percentage point change in homelessness claims: |  | |
| One year prior | -0.039 | |
|  | (0.050) | |
| Percentage Change in Local Authority Expenditure |  | |
| In contemporaneous year | -0.092^***^ | |
|  | (0.011) | |
| One year prior | -0.061^***^ | |
|  | (0.011) | |
| Percentage Change in Central Government Expenditure |  | |
| In contemporaneous year | 0.012 | |
|  | (0.017) | |
| One year prior | 0.067^***^ | |
|  | (0.013) | |
| Percentage Change in Gross Value Added per Capita | -0.0095 | |
|  | (0.017) | |
| Change in Unemployment Rate | -0.0048 | |
|  | (0.016) | |
| Number of Local Authority-Years | 1866 | |
| *R*^2^ | 0.112 | |

*Notes:* Standard errors estimated using fixed effects in parentheses clustered by Local Authority to reflect non-independence of sampling. Number of local authorities = 323.

^*^ *p* < 0.05, ^**^ *p* < 0.01, ^***^ *p* < 0.001

Web Table A8. Association of Previous Year Homelessness Rates with Local Authority Expenditure Changes in England, 323 Local Authorities, 2004-2012.

|  | Percentage Change in Local Authority Expenditure |
| --- | --- |
|  | (1) |
| Percentage point change in homelessness claims: |  |
| One year prior | -0.67^***^ |
|  | (0.096) |
| Percentage Change in Local Authority Expenditure |  |
| One year prior | -0.12^***^ |
|  | (0.028) |
| Percentage Change in Gross Value Added per Capita | -0.19^***^ |
|  | (0.046) |
| Change in Unemployment Rate | 0.21^***^ |
|  | (0.057) |
| Number of Local Authority-Years | 1904 |
| *R*^2^ | 0.081 |

*Notes:* Standard errors estimated using fixed effects in parentheses clustered by Local Authority to reflect non-independence of sampling. Number of local authorities = 323.

^*^ *p* < 0.05, ^**^ *p* < 0.01, ^***^ *p* < 0.001
