# Supplementary material for: The impact of economic downturns and budget cuts on homelessness claim rates across 323 local authorities in England, 2004–12
Source: J Public Health (Oxf). 2016 Oct 17;38(3):417–25. doi: 10.1093/pubmed/fdv126 (PMC5072163; doi:10.1093/pubmed/fdv126)
Supplement: Supplementary Data [file supp_fdv126_fdv126supp_AppendixB.docx]

**Web Appendix B: Detailed Methods and Definitions**

**Description of Local Authority Structure in England**

The local authorities comprise 201 Shire Districts, 36 Metropolitan Districts, 56 Unitary Authorities, and 32 London Boroughs and the City of London. In 2009, nine new Unitary Authorities were created, amalgamating 37 shire districts. To harmonise data for these shire districts, we re-calculated pre-2009 data based on the updated boundaries. The City of London and Isles of Scilly were excluded because the former has a unique role and status and the small resident population in the latter creates highly variable annual fluctuations. One shire district (Brentwood) was further excluded because of insufficient data on unemployment rates over the period.

**Statutory Homelessness in England**

To make a homelessness claim, households must meet specified criteria of citizenship or residency. To be accepted they must also demonstrate that they did not make themselves intentionally homeless, a local area connection to the local authority where they are applying, and that they are in “priority need.” Web Appendix Box B1 describes priority need and criteria for statutory homelessness acceptance.

In 2012, those accepted were primarily made up of households with children (64%) and households with a pregnant member (10%), with smaller proportions accepted on account of mental illness and physical disability (approximately 8% each) or for other reasons (i.e. old age, domestic violence) [^1^](#_ENREF_1). Households not accepted as homeless are primarily those that do not meet the criteria for homelessness or those not considered in priority need (i.e. single adults).

**Welfare Expenditure in England**

Web Figure B1 depicts the allocation of funds and breakdown of spending decisions across these two levels. Web Tables B2 and B3 provide detail on the spending categories specifically included in our analyses.

Briefly, England’s central government determines spending on welfare allowances, including Employment and Support Allowance, Job Seekers Allowance, Disability Living Allowances, Winter Fuel Allowance, Housing Benefit and Council Tax Benefit, among others. The actual magnitude of payments going to local authorities varies depending upon claimant counts and, in the case of Housing and Council Tax benefits, on council tax banding and local housing allowance rates. We obtained data on expenditure for these centrally allocated welfare allowances for each local authority from the UK Department of Work and Pensions, which is calculated based on claimant counts and average benefit amounts.

With funds received through block grants from central government, local revenue, and council tax, local authorities support the provision of education, housing, social care, transportation, cultural services, environmental services, planning and development, and central and other services. [^2^](#_ENREF_2) Except in the case of ring-fenced grants from central government, local authorities autonomously determine these funding decisions.

Web Table B1. Categories of decisions for homeless claims made to local authorities under Housing Act 1996 and Homelessness Act 2002.

| Total Homelessness Decisions | All claims made from households eligible for local authority homelessness assistance (i.e. not from abroad) |
| --- | --- |
| Accepted claims | Owed a statutory homeless owed a duty to be housed  until a settled housing solution is found by a local authority   - Homeless according to definition in Housing Act 1996 - Priority need group   - Homeless because of emergency (e.g. fire)   - Dependent children in household   - Member of household is pregnant   - Applicant aged 16-17; applicant 18-20 and formerly in care   - Vulnerable because of: old age, physical disability, mental illness/disability, other special reasons (e.g. drug or alcohol addiction, former asylum, served in HM Forces, domestic violence)Meet criteria for homelessness - Unintentionally homeless - Local area connection^1^ to local authority |
| Unaccepted claims | Not owed a duty to be housed by a local authority. Assessment made by local authority to assess housing need and provide advice and assistance to help find accommodation independently.   - Not in a priority need group; or - Found to be intentionally homeless; or - Not homeless |

*Notes:* ^1^ Criteria for local connection include past or present residence in local authority; employment in local authority; family association in local authority; or other special circumstances. Source [^3^](#_ENREF_3).

Web Table B2. Description of types of central government welfare expenditure

| Benefit | Description |
| --- | --- |
| Employment and Support Allowance and Related Benefits | Recipients of these benefits are those not intended to be available for employment due to disability or illness. Reflecting the phase-in of Employment and Support Allowance since 2008 to replace spending on Incapacity Benefits, Severe Disablement Allowance (for under Pension age), and Income Support due to Disability, this expenditure combines spending in all of these categories. |
| Job Seekers Allowance | Welfare benefit for individuals looking for work. |
| Disability Living Allowance | Benefit to support the living costs of people living with disabilities. |
| Housing Benefit | Benefit payment to help low income households pay for rent. Amount partially determined by housing rental rates in local authorities. |
| Discretionary Housing Payments | Payments to support temporary shortfalls for rent or start-up costs of tenancy for households qualifying for Housing Benefit payments. |
| Council Tax Benefit | Reductions for low income individuals paying council tax. |
| Pension Credit | Income support for low income people aged 65. |
| Pension-age Disability Support (Attendance Allowance and Severe Disablement for Pension age) | Income supports for severely disabled people aged 65 or over who need help with personal care. *Note:* Severe disablement allowance phased out from 2002, but past claimants continue to receive benefit. |
| Income Support for Lone Parents, Carers & Other Causes. | Income support payments for individuals with no or low income, working less than 16 hours a week, and meeting criteria of a carer, lone parent, or other special circumstances. |
| Carer’s Allowance | Benefit payment for individuals spending at least 35 hours a week caring for someone. |

*Notes:* Adapted from [^4^](#_ENREF_4)*.*

Web Table B3. Description of types of local authority welfare expenditure

| **Variable** | **Categories Included** | **Description of Services** |
| --- | --- | --- |
| Spending on Housing Services^1^ | --- | Provision of housing assistance and support for individuals at risk of homelessness  Spending through the Supporting People Program  Includes spending on housing strategy, housing advice, housing advances, support for housing renewal, and administration of housing benefits |
| Spending on Social Care for Children and Adults | --- | Children’s and families’ services – support; welfare; fostering; adoption  Services for older people – nursing; home; residential and day care; meals  Services for people with a physical disability; sensory impairment; learning disabilities or mental health needs  Supported employment  Youth justice – secure accommodation; youth offender teams  Asylum seeker |
| Spending on Other Services |  |  |
|  | Highways, Roads and, Transport | Highways – construction and maintenance of non-trunk roads and bridges |
|  |  | Street lighting |
|  |  | Traffic management and road safety; new line parking services |
|  |  | Airports; harbours and toll facilities |
|  |  | Public transport – concessionary fares; support to operators; co-ordination |
|  | Cultural services | Culture and heritage – archives; museums and galleries; public entertainment |
|  |  | Recreation and sport – sports development; indoor and outdoor sports and recreation facilities |
|  |  | Open spaces – national and community parks; countryside; allotments |
|  |  | Tourism – marketing and development; visitor information |
|  |  | Libraries and information services |
|  | Environmental services | Cemetery; cremation and mortuary services |
|  |  | Community safety; consumer protection; coast protection; trading standards |
|  |  | Environmental health – food safety; pollution & pest control; housing standards; public conveniences; licensing |
|  |  | Agricultural and fisheries services |
|  |  | Waste collection and disposal; street cleansing |
|  | Planning and development | Building and development control |
|  |  | Planning policy – including conservation and listed buildings |
|  |  | Environmental initiatives |
|  |  | Economic and community development |
|  | Central and other services | Court services – coroners etc  Local tax collection Registration of births; deaths and marriages  Emergency planning  Democratic representation  Local land charges  Corporate management  Elections – including registration of electors |

*Notes:* Adapted from [^2^](#_ENREF_2).

Web Figure B1. The structure of central and local government welfare expenditure in England.


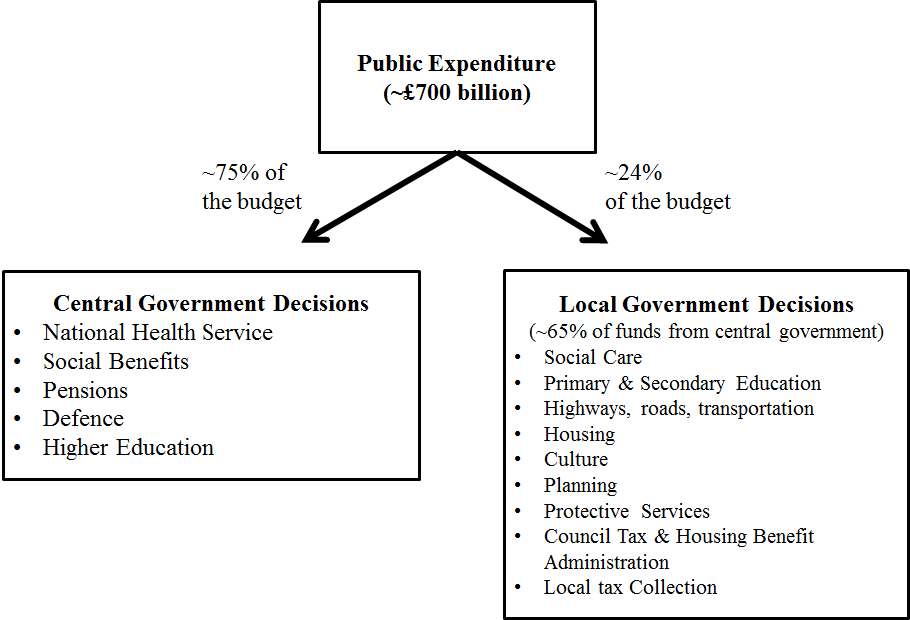


*Source:* UK Department of Communities and Local Government[^2^](#_ENREF_2).

**References**

1. Table 773: Reason for acceptance: Households accepted by local authorities as owed a main homelessness duty by priority need category, England 1998 to 2014. Gov.UK; 2014. https://[www.gov.uk/government/statistical-data-sets/live-tables-on-homelessness](http://www.gov.uk/government/statistical-data-sets/live-tables-on-homelessness). Accessed 19 December 2014.

2. Department of Communities and Local Government. *Local Government Financial Statistics England: No. 23 2013.* London2013.

3. Department of Communities and Local Government. Homelessness data: notes and definitions. 2014; https://[www.gov.uk/government/collections/homelessness-statistics](http://www.gov.uk/government/collections/homelessness-statistics). Accessed 15 June 2014, 2014.

4. Department of Work and Pensions. Benefits. 2014; https://[www.gov.uk/browse/benefits](http://www.gov.uk/browse/benefits). Accessed 15 May, 2014.
